# nifPred: Proteome-Wide Identification and Categorization of Nitrogen-Fixation Proteins of Diaztrophs Based on Composition-Transition-Distribution Features Using Support Vector Machine

**DOI:** 10.3389/fmicb.2018.01100

**Published:** 2018-05-29

**Authors:** Prabina K. Meher, Tanmaya K. Sahu, Jyotilipsa Mohanty, Shachi Gahoi, Supriya Purru, Monendra Grover, Atmakuri R. Rao

**Affiliations:** ^1^Division of Statistical Genetics, ICAR-Indian Agricultural Statistics Research Institute, New Delhi, India; ^2^Centre for Agricultural Bioinformatics, ICAR-Indian Agricultural Statistics Research Institute, New Delhi, India; ^3^Department of Bioinformatics, Orissa University of Agriculture and Technology, Bhubaneswar, India

**Keywords:** nitrogenase, diaztroph, Fe protein, Fe-Mo protein, biological nitrogen fixation, di-nitrogenase

## Abstract

As inorganic nitrogen compounds are essential for basic building blocks of life (e.g., nucleotides and amino acids), the role of biological nitrogen-fixation (BNF) is indispensible. All nitrogen fixing microbes rely on the same nitrogenase enzyme for nitrogen reduction, which is in fact an enzyme complex consists of as many as 20 genes. However, the occurrence of six genes viz., *nifB, nifD, nifE, nifH, nifK*, and *nifN* has been proposed to be essential for a functional nitrogenase enzyme. Therefore, identification of these genes is important to understand the mechanism of BNF as well as to explore the possibilities for improving BNF from agricultural sustainability point of view. Further, though the computational tools are available for the annotation and phylogenetic analysis of *nifH* gene sequences alone, to the best of our knowledge no tool is available for the computational prediction of the above mentioned six categories of nitrogen-fixation (nif) genes or proteins. Thus, we proposed an approach, which is first of its kind for the computational identification of nif proteins encoded by the six categories of nif genes. Sequence-derived features were employed to map the input sequences into vectors of numeric observations that were subsequently fed to the support vector machine as input. Two types of classifier were constructed: (i) a binary classifier for classification of nif and non-nitrogen-fixation (non-nif) proteins, and (ii) a multi-class classifier for classification of six categories of nif proteins. Higher accuracies were observed for the combination of composition-transition-distribution (CTD) feature set and radial kernel, as compared to the other feature-kernel combinations. The overall accuracies were observed >90% in both binary and multi-class classifications. The developed approach further achieved >92% accuracy, while evaluated with blind (independent) test datasets. The developed approach also produced higher accuracy in identifying nif proteins, while evaluated using proteome-wide datasets of several species. Furthermore, we established a prediction server nifPred (http://webapp.cabgrid.res.in/nifPred) to assist the scientific community for proteome-wide identification of six categories of nif proteins. Besides, the source code of nifPred is also available at https://github.com/PrabinaMeher/nifPred. The developed web server is expected to supplement the transcriptional profiling and comparative genomics studies for the identification and functional annotation of genes related to BNF.

## Introduction

Atmospheric nitrogen is the main natural source of nitrogen, where the fixing of di-nitrogen gas (N_2_) into a more active form (e.g., ammonia) is necessary before nitrogen molecules can be consumed by any organism (Schimpl et al., 2003). In this regard, biological-nitrogen-fixation (BNF) is a key biogeochemical process (Ward et al., 2007), which contributes maximum percentage to the total nitrogen fixation (Seefeldt et al., 2009). Besides its role in the global nitrogen cycle, BNF is also directly proportional to the agricultural sustainability (Bohlool et al., 1992). Due to unavailability of fixed nitrogen in soil, synthetic nitrogenous fertilizers (nitrogen fixation through Haber-Bosch process) are applied as a common agricultural practice to increase the production, which has hazardous impact on plant, animal, and human health (Saikia and Jain, 2007).

The process of BNF is carried out by nitrogen fixing microbes, also called diaztrophs. All the diaztrophs rely on the same nitrogenase enzyme for nitrogen reduction. The nitrogenase is rather an enzyme complex consists of two metallo proteins viz., iron-molybdenum (FeMo) protein that performs the reduction and iron (Fe) protein which provides electron (Hamilton et al., 2011; Frank, 2014). Though nitrogenase enzyme may contain as many as 20 genes, the main structural genes are *nifD, nifH*, and *nifK*. The *nifH* is the structural gene for Fe protein, whereas *nifD* and *nifK* are the respective structural genes for α and β subunits of FeMo protein. In addition to these three coding genes, co-occurrences of three more genes viz., *nifE, nifN*, and *nifB* are assumed to be essential for a functional nitrogenase enzyme in diaztrophs (Dos Santos et al., 2012). The *nifE, nifN*, and *nifB* encoded proteins play essential role in the assembly and incorporation of Fe and Mo atoms into the nitrogenase subunits. In particular, the product of *nifB* gene catalyzes the formation of a FeMo cofactor precussor called the B-cofactor, whereas the *nifE* and *nifN* function as scaffolds for Fe-Mo cofactor assembly (Roll et al., 1995).

Most of the earlier studies have focussed either on the annotation of *nifH* sequences or on the phylogenetic distribution of diaztrophs by using *nifH* sequences as markers (Mondal et al., 2008). In particular, Gaby and Buckley (2014) designed a database that contains 32954 aligned nitrogenase *nifH* sequences that facilitates phylogenetic and evolutionary studies of nitrogen-fixing microorganism. Further, Heller et al. (2014) have developed a software pipeline, *ARBitrator*, for retrieving auto-curated *nifH* sequences from Genebank. Another computational method based on classification and regression trees (CART) was developed by Frank et al. (2016) for the annotation of *nifH* gene sequences, where the classification of *nifH* protein sequences into different phylogenetic clusters was performed. Furthermore, to the best of our knowledge, no tool is available in literature for the computational prediction of nif proteins encoded by the above mentioned six categories of nif genes. Though comparative genomic analysis (Dos Santos et al., 2012; Xie et al., 2014; Inoue et al., 2015) and transcriptional profiling (Yan et al., 2010; Orr et al., 2011; Sarkar and Reinhold-Hurek, 2014) studies have been carried out for identifying the nif genes, establishment of a prediction tool for the computational identification of nif genes would further add on to accelerate the research in the area of BNF. Besides identifying nif genes, the computational tool can also be useful to identify and categorize potential diaztrophs. Moreover, identifying these genes would further help in understanding the mechanism of BNF. Although transcriptome and comparative genomics studies are useful for identifying these genes, they are species-specific and sometime computationally expensive as well. Thus, developing a computational tool would certainly be helpful to identify the nif genes in highthroughput sequence data.

In view of above prospects, here we made an attempt to establish a novel predictor for computational identification of nif proteins encoded by *nifB, nifD, nifE, nifH, nifK*, and *nifN* genes. The sequences were first mapped into numeric feature vectors and then the encoded vectors were supplied as input to machine learning classsifier. Two types of classifiers were constructed. In the first type, binary classifier was constructed to classify nif and non-nif proteins, and in the second type, multi-class classifier was built for classification of six categories of nif proteins. Both types of classifiers were developed using the sequence-based features of protein sequences. We have further established a web server for proteome-wide identification of proteins encoded by the considered six categories of nif genes. The developed approach is believed to supplement the existing efforts in identifying and annotating the nif genes.

## Materials and methods

### Collection and processing of dataset

For binary classification, two classes of datasets are required viz., positive (nif) and negative (non-nif). In this study, protein sequences encoded by *nifB, nifD, nifE, nifH, nifK*, and *nifN* genes were considered as the positive dataset. These protein sequences (102 *nifH*, 95 *nifD*, 100 *nifK*, 88 *nifE*, 86 *nifN*, and 81 *nifB*) were collected from the 82 diaztrophs reported in Dos Santos et al. (2012). The dataset was then processed to remove the sequences having non-standard amino acid residues. Further, the negative dataset was constructed by using all other protein sequences of the above mentioned 82 species which were obtained from Uniprot (http://www.uniprot.org/). The sequences with non-standard residues were also removed from the negative dataset. Furthermore, to avoid homologous bias in both positive and negative datasets, redundant sequences at different levels of pair-wise sequence identities were removed using CD-HIT (Fu et al., 2012). In particular, three different positive sets were prepared having sequences with <60%, <70%, and <90% pair-wise sequence identities. The number of instances for the positive sets at three different levels of pair-wise sequence identities can be seen from Table 1. For the negative class, one dataset of 135525 sequences was prepared in which each sequence was <40% identical to any other sequences.

**Table 1:** Summary of the collected dataset with different percentage of sequence identity.

**Pair-wise sequence identity (%)**	**#Sequence**						**Total**
	***nifH***	***nifD***	***nifK***	***nifE***	***nifN***	***nifB***	
60	8	13	24	20	41	25	116
70	13	24	37	39	57	38	193
90	59	72	86	80	80	74	438

*Three different datasets were prepared, where the sequences having higher pair-wise sequence identities than the considered threshold were excluded using CD-HIT program. The number of sequences at different level of identities show that the nifH sequences are more conserved and nifN sequences are least conserved among six categories of protein sequences. The last column represents the total number of sequences in the dataset at different level of pair-wise sequence identity*.

### Feature generation

Feature representation plays a significant role on the prediction accuracy of machine learning-based predictor/classifier. Moreover, the amino acid sequences are required to be transformed into vectors containing numeric observations before being supplied as input to machine learning techniques (Zhang et al., 2006). In the present study, we used six different sequence-based features to map the amino acid sequences into vectors of numeric observations. The features are compositions of amino acids (AAC; Bhasin and Raghava, 2004; Cai and Chou, 2006), compositions of di-peptides (DPC; Bhasin and Raghava, 2004; Meher et al., 2017), pseudo amino acid compositions (PseAAC; Chou, 2001), composition-transition-distribution (CTD; Dubchak et al., 1995; Cai et al., 2003; Govindan and Nair, 2011), gap-pair compositions (GPC; Yu et al., 2006), and auto-correlation function (ACF; Liu and Chou, 1998; Zhang et al., 1998). Succinct descriptions about computation of the above mentioned features are given in the following sub-sections.

#### Amino acid composition (AAC)

AAC is the simplest and most widely used feature for representing the protein sequences. It is nothing but the proportions of amino acid residues present in the sequence. Based on AAC, every protein sequence can be converted to a vector of 20 numeric observations. For a protein sequence with *N* residues, AAC for the *i*^th^ amino acid can be computed as *AAC*(*i*) = *f*_*i*_/*N*, where *i* = 1, 2, …, 20 and *f*_*i*_ indicates the number of times *i*^th^ amino acid present in the sequence.

#### Di-peptide composition (DPC)

Unlike AAC, DPC takes the ordering effects of amino acid residues within a short range into consideration (Ding et al., 2004). Anticipating improvement in accuracy by accounting the local-ordering of residues, DPC were considered as features. For any di-peptide *M*_*j*_, DPC can be computed as *DPC*(*j*) = *M*_*j*_/(*N* − 1), where *j* = 1, 2,…, 400 and *N* denotes the sequence length. Using DPC, each protein sequence can be transformed into a 400-dimensional numeric vector.

#### Gap-pair composition (GPC)

For a given sequence with *N* amino acid residues, GPC for amino acid pair (*i, j)* with G-gap can be obtained as $f_{G}\left(i,j\right)=\frac{D_{G}\left(i,j\right)}{\left(N-G-1\right)}$, where *i, j* = 1, 2, …, 20 and *D*_*G*_(*i, j*) is the number of times the amino acid pair (*i, j)* appears in the sequence. Using GPC features, every amino acid sequence can be encapsulated with a numeric vector of 400 elements. Presently, we used 1 gap-pair (GPC-1) and 2 gap-pair (GPC-2) compositions as features. More clearly, for GPC-1 and GPC-2, the features are nothing but the proportions of amino acid pairs (*i, j)* separated by one residue (*ixj*), and two residues (*ixxj*) respectively, where *x* denotes any residue.

#### Pseudo amino acid composition (PseAAC)

The idea of PseAAC was brought up by Chou (2001). The PseAAC not only takes into account the sequence-ordering information within a local range but also the global sequence-ordering effects. This feature has been proven effective in many protein-related classifications (Wang et al., 2010). Using PseAAC, every protein sequence can be encoded to a (20+d)-dimension vector of numeric observations for d-tier correlation structure. In the present study, 1^st^-tier correlation was only used to extract PseAAC features. For more details on PseAAC, earlier studies (Chou, 2005, 2009) can be referred.

#### Composition-transition-distribution (CTD)

Dubchak et al. (1995) introduced the concept of CTD feature while making the prediction for different classes of protein folding. Since its introduction, the CTD feature has been successfully employed in many functional and structural related studies of proteins (Govindan and Nair, 2011). In CTD, C (composition) stands for the compositions of amino acids, T (transition) represents the percentage with which frequency of amino acids with specific properties is followed by amino acids with other properties and D (distribution) determines the length of the sequence within which the 1^st^ as well as 25, 50, and 75 percents of amino acids of certain characteristics are located. With CTD feature, each sequence of *N* amino acid residues can be encoded to a numeric vector of $N+\left\{N*\frac{N-1}{2}\right\}+\left(N*5\right)$ elements.

#### Auto-correlation function (ACF)

Auto-correlation takes into account the dependencies among sequence features, which are computed by taking the distribution of amino acid properties into account. Here, the ACF-based features were computed by considering all 531 amino acid properties obtained from *AAindex* database (Kawashima and Kanehisa, 2000). Using ACF features, every sequence can be encoded to a (531^*^n)-dimensional vector of numeric observations, for n^th^ order autocorrelation. Here, we considered the 1^st^ order autocorrelation only, because with higher order number of features will be very large.

### Support vector machine (SVM) classifier

In the present work, SVM (Vapnik, 2000) was employed for classification purpose. Since SVM is non-parametric in nature with a strong statistical background (based on the statistical principle of structural risk minimization), it has been efficiently employed in numerous biological studies including bioinformatics (Guo et al., 2014; Chen et al., 2015; Liu et al., 2015) and computational biology (Chen et al., 2013, 2016; Lin et al., 2014; Qiu et al., 2014). The ability of SVM to handle large and noisy input dataset further makes it an attractive machine learning tool for computational studies. The performance of SVM highly depends upon the type of kernel functions used. The kernel function maps the input dataset into high-dimensional feature space, where the optimal separating hyper plane linearly separates the observations of different groups. By using a subset of 100 nif and 100 non-nif protein sequences, four basic kernels (radial, sigmoid, polynomial, and linear) with default parameters were initially employed to choose the best fitted kernel (for which highest accuracy was obtained) that was used in the subsequent analysis. In this work, two different types of classification task were carried out: (i) a binary classifier to classify nif and non-nif proteins, and (ii) a multi-class classifier to distinguish six categories of nif proteins from each other. The *svm* function available in “e1071” package (Meyer et al., 2015) of R-statistical software (R Development Core Team, 2012) was run for implementing the SVM model.

### Assessment through cross-validation

For assessing the performance of newly established machine learning predictor, cross-validation (CV) analysis is essential (Henderson et al., 1996). More often, three different forms of cross-validation viz., K-fold CV, jackknife-validation, and validation with independent test set are preferred (Chou, 2011). In fact, we used all the three CV techniques in our study. The five-fold CV was employed for assessing the performance of binary classifier, whereas the jackknife-validation was used for evaluating the multi-class classifier. For five-fold CV, entire input dataset was divided at random into 5 equal-sized sets containing approximately same number of observations from both nif and non-nif categories where in each fold four sets constituted the training set and the remaining one was used as test set. This procedure was repeated for five times in such a fashion that every set got exactly one chance to be tested by the respective trained model. In the jackknife-validation, the entire dataset was partitioned into as many as training and test sets whose number was same as the size of the dataset. In every step of the jackknife validation, all the observations except one constituted the training set and the remaining one observation was used as the test instance. Besides, the developed predictor was also evaluated with independent test datasets that were neither used in model building nor in testing the model.

### Classification using balanced dataset

As the number of protein sequences in non-nif category are much larger than that of nif category, the dataset is highly unbalanced. With unbalanced dataset, machine learning-based classifier may produce results biased toward the major class (having large number of sequences than the other class). Therefore, the binary classification was carried out using balanced dataset consisting of approximately equal number of instances from both nif and non-nif classes, where the non-nif sequences were drawn at random from the whole non-nif dataset. Since the size of the negative (non-nif) dataset is large, using one set of random observations may not be adequate to assess the generalized performance of the classification model. Therefore, the binary classifier was evaluated with 100 random sample sets, where in each set almost equal number of instances from both nif and non-nif classes were present. Moreover, five-fold cross validation analysis was performed in each sample set and the performance metrics for the binary classifier were measured by taking average over the 100 sample sets. As the performance was measured using jackknife validation, problem of unbalanced-ness was not considered for the multi-class classification.

### Evaluating the performance

We considered the sensitivity (Sn), specificity (Sp), Precision (Pre), Matthew's correlation coefficient (MCC), and accuracy (Ac) to measure the prediction accuracy of the proposed computational model, because the same metrics have been used for assessing the prediction accuracies of machine learning-based predictors in numerous studies (Chou, 2002; Jia et al., 2016a,b; Liu and Long, 2016; Liu et al., 2016). Instead of using conventional formulae to define above mentioned metrics, Chen et al. (2013) redefined these metrics with different notations to make them easier to understand and more intuitive. The redefined formulae of the metrics are given by

$$\left\{ \begin{array}{l} Sn=\left(1-\frac{N_{n}^{p}}{N^{p}}\right);Sp=\left(1-\frac{N_{p}^{n}}{N^{n}}\right);Ac=\left(1-\frac{N_{n}^{p}+N_{p}^{n}}{N^{p}+N^{n}}\right)\\ \;MCC=\frac{1-\left(\frac{N_{n}^{p}}{N^{p}}+\frac{N_{p}^{n}}{N^{n}}\right)}{\sqrt{\left(1+\frac{N_{p}^{n}-N_{n}^{p}}{N^{p}})(1+\frac{N_{n}^{p}-N_{p}^{n}}{N^{n}}\right)}};Pre=1-\frac{N_{p}^{n}}{N^{p}-N_{n}^{p}+N_{p}^{n}}, \end{array}\right.$$

where *N*^*p*^, *N*^*n*^, $N_{p}^{n},$ and $N_{n}^{p}$ denote the number of nif proteins observed, non-nif proteins observed, non-nif proteins misclassified as nif proteins, and nif proteins misclassified as non-nif proteins respectively.

### Evaluation using other supervised learning techniques

Besides SVM, a number of supervised learning techniques are also available in literature. Out of those, Boosting (Drucker et al., 1994), Artificial neural network (ANN; Haykin, 1999), Bagging (Breiman, 1994), *k*-nearest neighbor (*k*NN; Hand et al., 2001), Naive Bayes (NB; Mitchell, 1997), and Random forest (RF; Breiman, 2001) are the important ones. Therefore, performances of these approaches were also evaluated and compared with that of SVM (with best combination of feature set and kernel function for which highest accuracy was obtained). The performances were assessed using the same 100 sample sets as mentioned in the subsection “Classification using balanced dataset,” where in each sample set approximately same number of nif and non-nif sequences were present. The *knn, bagging, ada, NaiveBayes, randomForest*, and *mlp* functions of the respective R-packages “klaR” (Weihs et al., 2005), “class” (Venables and Ripley, 2002), “ada” (Culp et al., 2016), “ipred” (Peters and Hothorn, 2013), “randomForest” (Liaw and Wiener, 2002), and “RSNNS” (Bergmeir and Benitez, 2012) were used to execute the *k*NN, Bagging, Boosting, NB, RF, and ANN classification models respectively.

### Comparison with blast algorithms

The highly similar sequences are believed to share similar structure and function, and this approach has been widely adopted in the past for protein and peptide prediction (Frank and Sippl, 2008). Keeping this in mind, two blast algorithms viz., BlastP (Altschul et al., 1990) and PSI-Blast (Altschul et al., 1997) were employed for the classification of nif and non-nif proteins, and their performances were compared with that of proposed approach as well. The performances of the blast algorithms were assessed using five-fold CV procedure. For CV, offline version of blast from NCBI was first installed in a local server. Then the BlastP and PSI-Blast algorithms were executed, where in each fold the training set was designated as the database and the respective test set as query. Every query sequence was matched against the locally created databases using blast search. The query sequence was predicted as nif if significant similarity was found with nif proteins, else non-nif if found with non-nif proteins.

### Comparison with hidden markov model (HMM)

The performance of the proposed model was also compared with that of HMM. The HMM analysis was performed with the help of standalone version of HMMER 3.1b2 (Johnson et al., 2010). In each fold of the five-fold CV, the HMM profile was created using the nif category of the training set by employing the module *hmmbuild*. The sequences of the corresponding query set having instances from both nif and non-nif classes were then searched against the respective constructed HMM profile using the module *hmmsearch* in the HMMER suite.

### Evaluating performance with blind datasets

**Figure 1:**
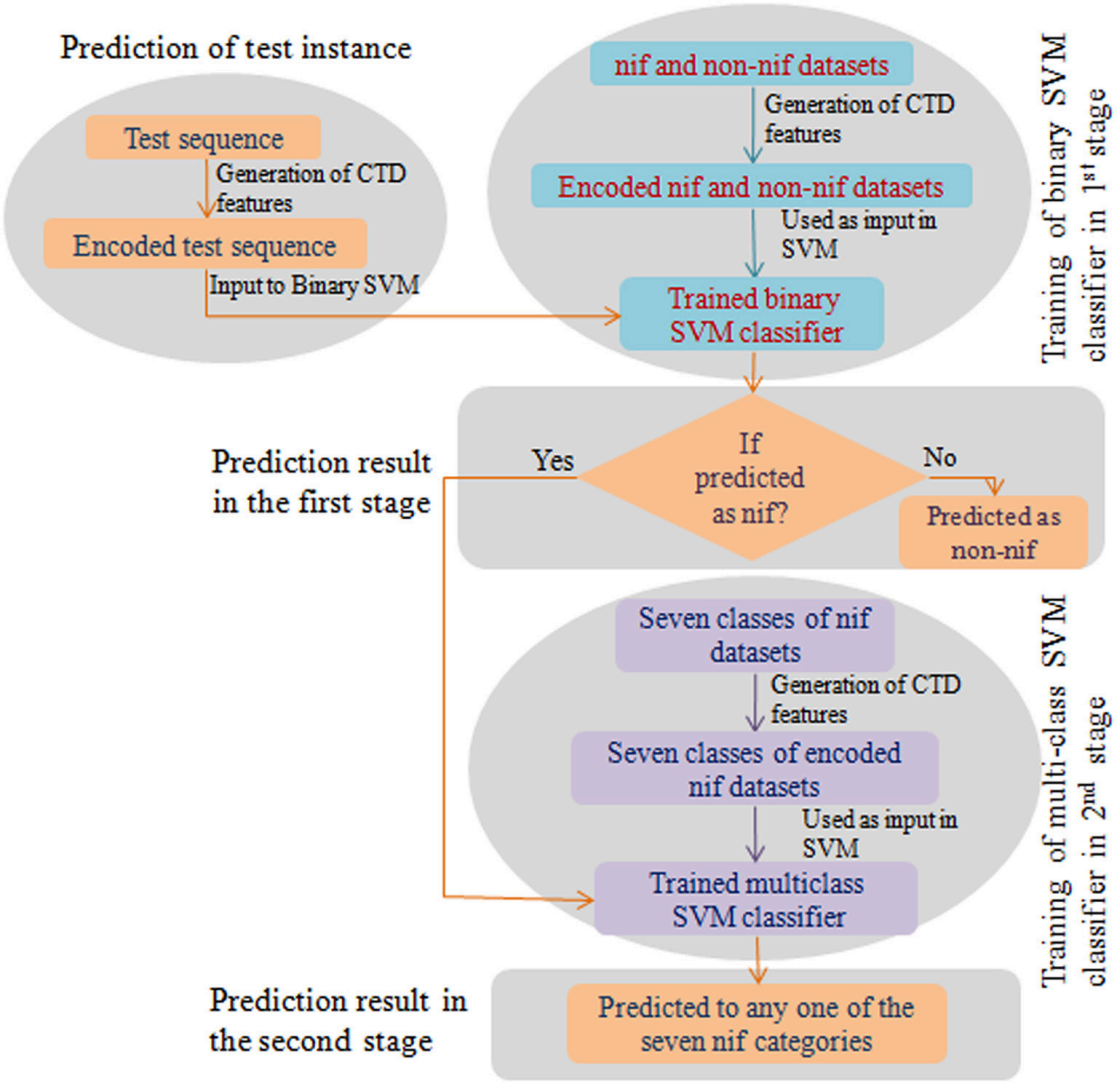
Flow diagram of the prediction process. This diagram shows the steps involved in construction of a binary and multiclass classifiers and the prediction of the test instance in two stages.

To weigh up the generalized performance, the prediction accuracy of the developed computational model was tested with two different blind (independent) datasets. The first one (Test set-I) consists of nif protein sequences from 67 diaztrophs that has been predicted by Dos Santos et al. (2012). Since the number of sequences in Test set-I were less and confined to a small number of species, we further collected the nif protein sequences from InterPro (https://www.ebi.ac.uk/interpro/) database and is designated as Test set-II. The datasets were also processed to remove the duplicate sequences as well as the sequences with non-standard amino acid residues. The number of sequences obtained after processing are given in Supplementary Table S1. Further, prediction for the test instances was made in two stages. In the first stage, protein sequences were classified as nif or non-nif, and those sequences classified as nif were only subjected to the second stage where they classified into any one of the considered six categories of nif proteins. A flow diagram with the steps involved for prediction of test instance is shown in Figure 1.

### Proteome-wide identification

To assess the performance of the developed approach for identifying nif proteins at whole genome level, prediction was made using proteome-wide datasets of 10 nitrogen fixing and 10 non-fixing strains of genus *Paenibacillus*. We considered this dataset because it has been used by Xie et al. (2014). The protein sequences for these strains were collected from NCBI (https://www.ncbi.nlm.nih.gov/), summary of which is provided in Supplementary Table S2.

### Development of prediction server

A web server was also established for proteome-wide identification of nif proteins encoded by the six types of nif genes. The front-end of the server was designed with hypertext mark-up language (HTML), where an in-house R-script was run at the back end using hypertext pre-processor (PHP) for the prediction task. Besides, Java script was used for client side customizations. Provisions are made to upload the files as well as to paste the sequences in the text area. The sequences with standard amino acid residues need to be supplied in FASTA format for making prediction using the developed server.

## Results

### Kernel and feature analysis

**Figure 2:**
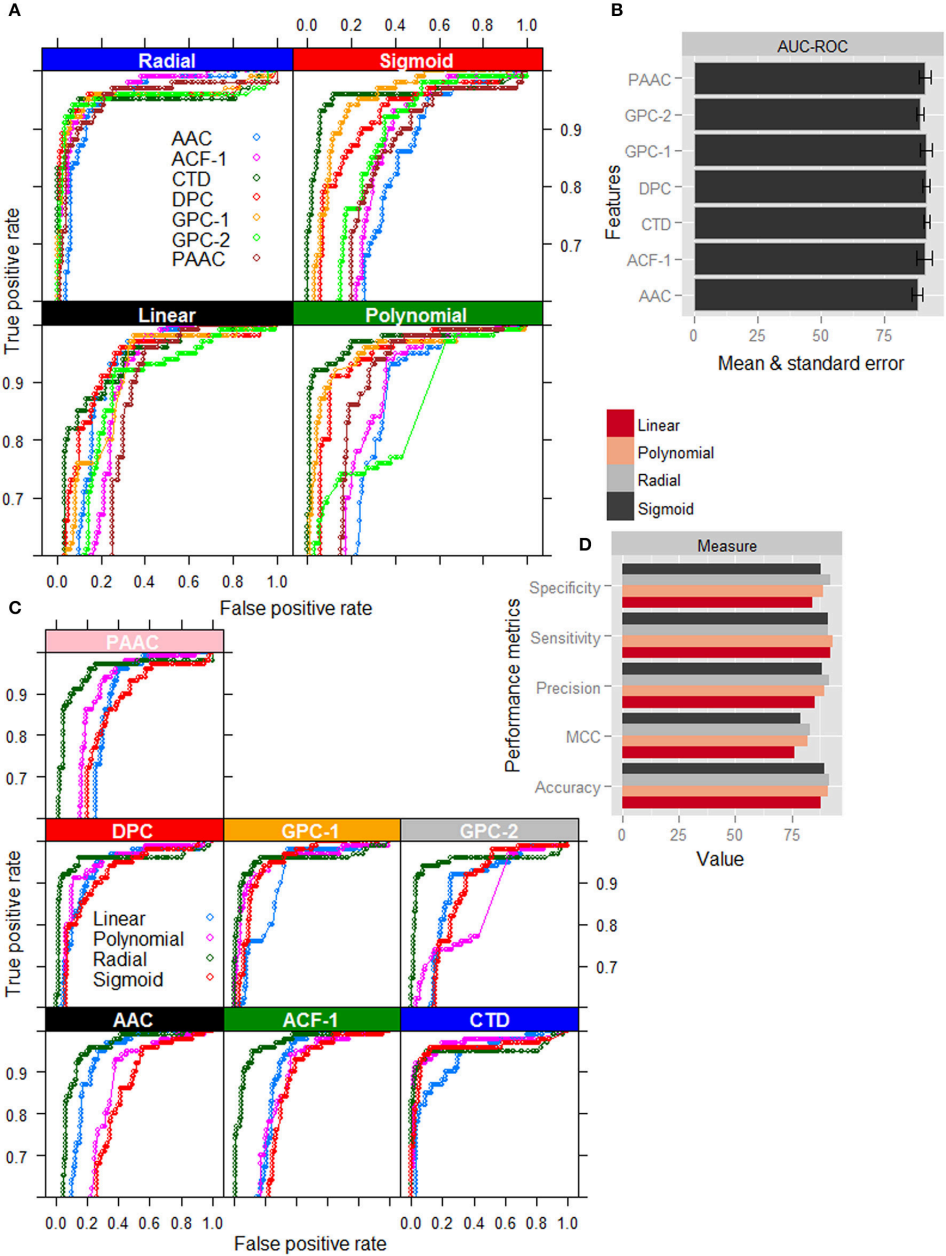
Graphical representation of performance metrics for different feature-kernel combinations. **(A)** ROC curves of four different kernels of SVM for classification of nitrogen-fixation (nif) and non-nitrogen-fixation (non-nif) proteins with seven different feature sets. **(B)** Bar plots of AUC-ROC for radial kernel of SVM with different combination of features. **(C)** ROC curves for different feature sets with respect to classification of nif and non-nif proteins using four different kernels of SVM. **(D)** Bar plots of performance metrics for four different kernels with CTD feature set. The figures show that the combination of radial kernel and CTD feature set is better than the other feature-kernel combinations for classification of nif and non-nif proteins.

Except radial kernel, it is clearly seen that the classification accuracy in terms of ROC curve (Figure 2A) is highest for the CTD among all the feature sets. On the other hand, for the radial kernel, though the accuracies in terms of AUC-ROC (Figure 2B) are observed almost same for GPC-1, DPC, CTD, and ACF-1 feature sets, standard error is seen to be lowest for CTD feature set. Besides, the number of features in CTD (310) is also less than that of DPC (400) and ACF-1 (531) feature sets. It is further observed that except CTD feature set the classification accuracies are higher for the radial kernel than that of other three kernels in AAC, DPC, PseAAC, GPC, and ACF feature sets. On the other hand, in case of CTD features, though the ROC curves for polynomial, sigmoid, and radial kernels (Figure 2C) are seen very close to each other, performance metrics for radial kernel are observed little higher followed by polynomial and sigmoid kernels (Figure 2D). Furthermore, it is observed that the radial kernel is more robust to different feature sets as compared to other three kernels (Figure 2A). From the above analysis, it is inferred that the accuracies under the combination of radial kernel and CTD feature set are higher than that of other feature-kernel combinations, and hence the same combination is followed in the subsequent analysis.

### Performance analysis of supervised learning techniques

**Figure 3:**
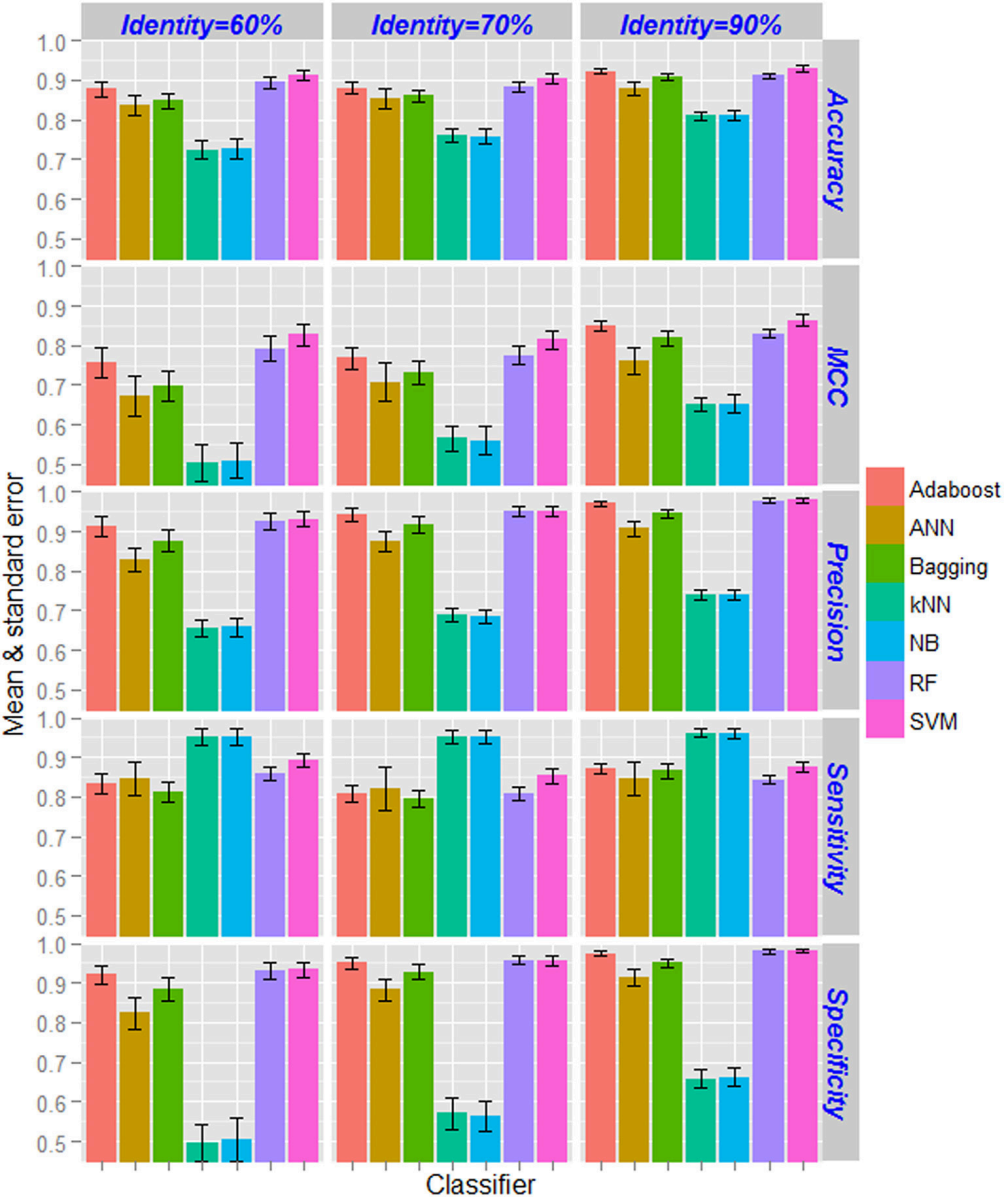
Bar diagrams of the estimates of performance metrics for different supervised learning techniques. The performance of SVM was compared with other six machine learning approaches with respect to classification of nif and non-nif proteins with CTD feature sets. Classification accuracies increased with increase in pair-wise sequence identity level in the positive dataset. The accuracies of kNN and NB classifiers are observed to be lowest, whereas highest accuracies are observed for SVM followed by RF classifier. The performance metrics of SVM are also found to be more stable (less standard error) as compared to the other classifiers.

Based on the CTD features, performance metrics of SVM (with radial kernel) and other supervised learning techniques computed by taking average over 100 sample sets (as mentioned in section Classification Using Balanced Dataset) as well as five-fold in each set are shown in Figure 3. From the figure, highest and lowest values of performance metrics can be seen for the dataset having <90% and <60% pair-wise sequence identities respectively. Similarly, the accuracies are also seen to be more stable (less standard error) for the dataset with nif protein sequences at <90% pair-wise identity, and least stable with <60% pair-wise identity. As far as the accuracies of supervised learning approaches are accounted, kNN and NB achieved almost same accuracy and is lowest among all the classifiers. On the other hand, higher accuracies are obtained for SVM followed by RF. It is further observed that the accuracies are least stable for ANN, and most stable for SVM and RF. Interestingly, accuracies for SVM are observed >0.9 in all the three datasets. Though specificities of RF are seen at par with that of SVM, higher accuracies are observed for SVM in terms of other metrics. Overall, the performance metrics of SVM are observed better than that of other machine learning classifiers.

### Ono-to-one prediction analysis

**Figure 4:**
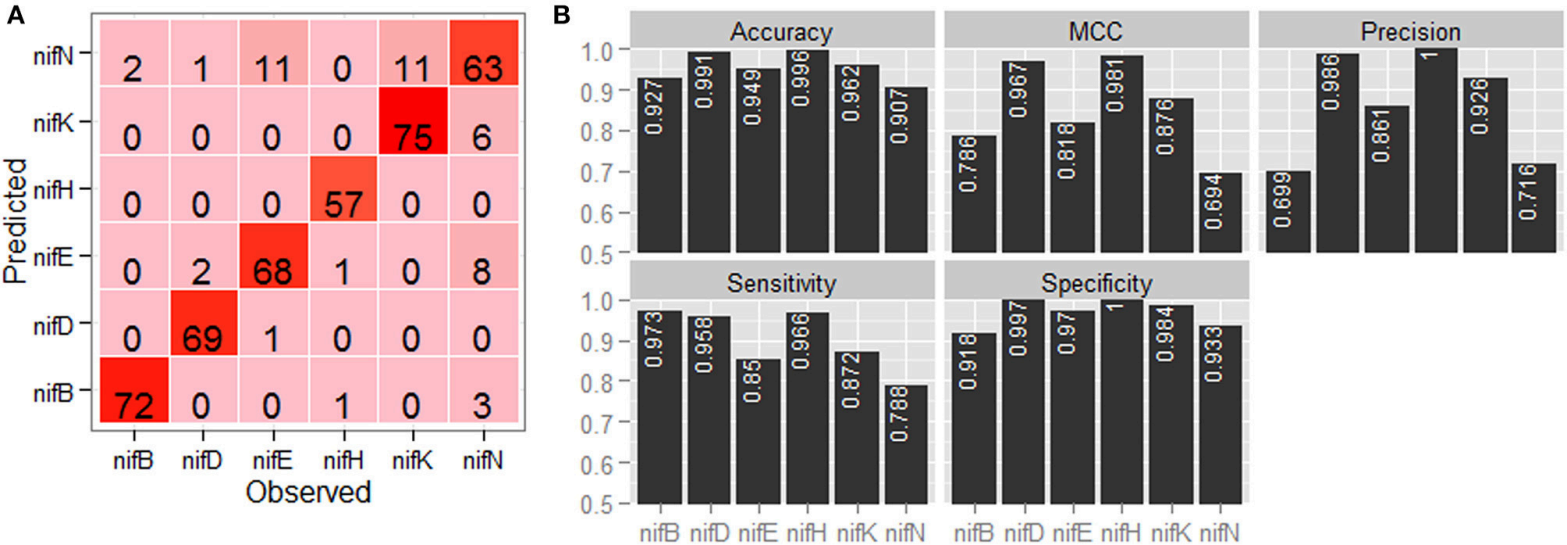
Graphical representation of the prediction accuracy of the developed approach under jackknife validation. **(A)** Confusion matrix and **(B)** bar plots of performance metrics for classification of six categories of nitrogen fixation-proteins using jackknife validation technique. From the confusion matrix (color from light to dark represents lower to higher numbers), it is seen that the protein sequences are mostly misclassified in *nifN* category, whereas no sequence is misclassified in *nifH* category. From the performance metrics, accuracies in discriminating *nifH, nifD*, and *nifK* from rest of the sequences are found higher among the six categories of protein sequences.

Confusion matrix with regard to the classification of six categories of nif proteins by employing jackknife validation is shown in Figure 4A. It is observed that the sequences are mostly misclassified into *nifN* category, whereas none of the sequences are misclassified into *nifH* category and only one sequence is misclassified into *nifD* category. From the performance metrics (Figure 4B), it is further observed that the accuracies in discriminating *nifH* and *nifD* from other categories are higher, whereas the accuracy is lowest for discriminating *nifN* from other categories. In particular, the accuracy, precision and MCC for *nifH* and *nifD* are seen >0.99, >0.98, and >0.96 respectively.

### Comparative analysis with blast algorithm

Based on 438 nif (Table 1) and 438 non-nif protein sequences (randomly drawn from the available non-nif sequences), the developed computational method was further compared with homology-based algorithms BlastP and PSI-Blast. Blast algorithms were first executed with three different e-values e.g., 0.1, 1, and 10, but no hits were found for most of the sequences belonging to the non-nif category for the e-values 0.1 and 1. Thus, we could not perform blast with lesser e-values, and the blast results are only reported here for e-value 10. The performance metrics averaged over the five-folds of CV are given in Table 2. From the table, the number of false positives (falsely predicted in nif category) in BlastP and PSI-Blast are seen to be much higher (low specificity) than that of proposed one. On the other hand, the number of false negatives are higher for the proposed one. Nonetheless, performance metrics of the proposed approach in terms of overall accuracy, precison and MCC are observed much higher than that of blast algorithms. In particular, overall accuracy of the proposed approach is observed ~0.94, which is >25% higher than that of BlastP and PSI-Blast. Similar trends are also observed for precision and MCC.

**Table 2:** Performance metrics of the proposed approach and blast algorithms.

**Approach**	**Sensitivity**	**Specificity**	**Accuracy**	**Precision**	**MCC**
Proposed	0.887	0.993	0.940	0.992	0.885
BlastP	0.995	0.538	0.767	0.683	0.600
PSI-Blast	0.995	0.545	0.770	0.686	0.605

*The performance of the developed method was compared with that of BlastP and PSI-Blast with respect to the classification of nitrogen-fixation (nif) and non-nitrogen-fixation (non-nif) proteins, where the performances were measured over the 5-folds of the cross-validation. The blast algorithms are observed highly biased toward the nif category. Though the sensitivity of the proposed approach is seen to be less than that of BlastP and PSI-Blast, specificity is observed much higher for the proposed approach. Nevertheless, the overall accuracy, precision and MCC for the proposed approach are observed much higher than that of blast algorithms*.

### Comparative analysis with HMM

The performance of HMM was assessed using the same dataset that was used to evaluate the performance of blast algorithms, with two different e-values 1 and 10. The performance metrics averaged over the 5-folds of CV are given in Table 3. From the table it can be seen that all the performance metrics of the proposed approach are higher than that of HMM for both e-values. In particular, with lesser e-value, though the number of false positives are seen to be declined (high precision), number of true positives are also seen to be declined (less sensitivity). However, the overall accuracy at e-value 1 (0.907) is observed to be higher than that of e-value 10 (0.845). Nevertheless, the overall accuracy of the proposed approach is found to be ~3% and ~10% higher than that of HMM with e-values 1 and 10, respectively. In comparison to blast, the overall accuracies of the HMM (Table 3) are found to be much higher than that of blast algorithms (Table 2).

**Table 3:** Performance metrics of the proposed approach and hidden Markov model (HMM).

**Approach**	**e-value**	**Sensitivity**	**Specificity**	**Accuracy**	**Precision**	**MCC**
HMM	1	0.834	0.979	0.907	0.980	0.841
	10	0.876	0.814	0.845	0.813	0.709
Proposed	NA	0.887	0.993	0.94	0.992	0.885

*The performance of the developed approach was also compared with that of HMM for classification of nitrogen-fixation (nif) and non-nitrogen-fixation (non-nif) proteins. In terms of all the performance metrics, the developed approach achieved higher accuracies than that of HMM. NA, Not applicable*.

### Analysis of the independent test set prediction

**Figure 5:**
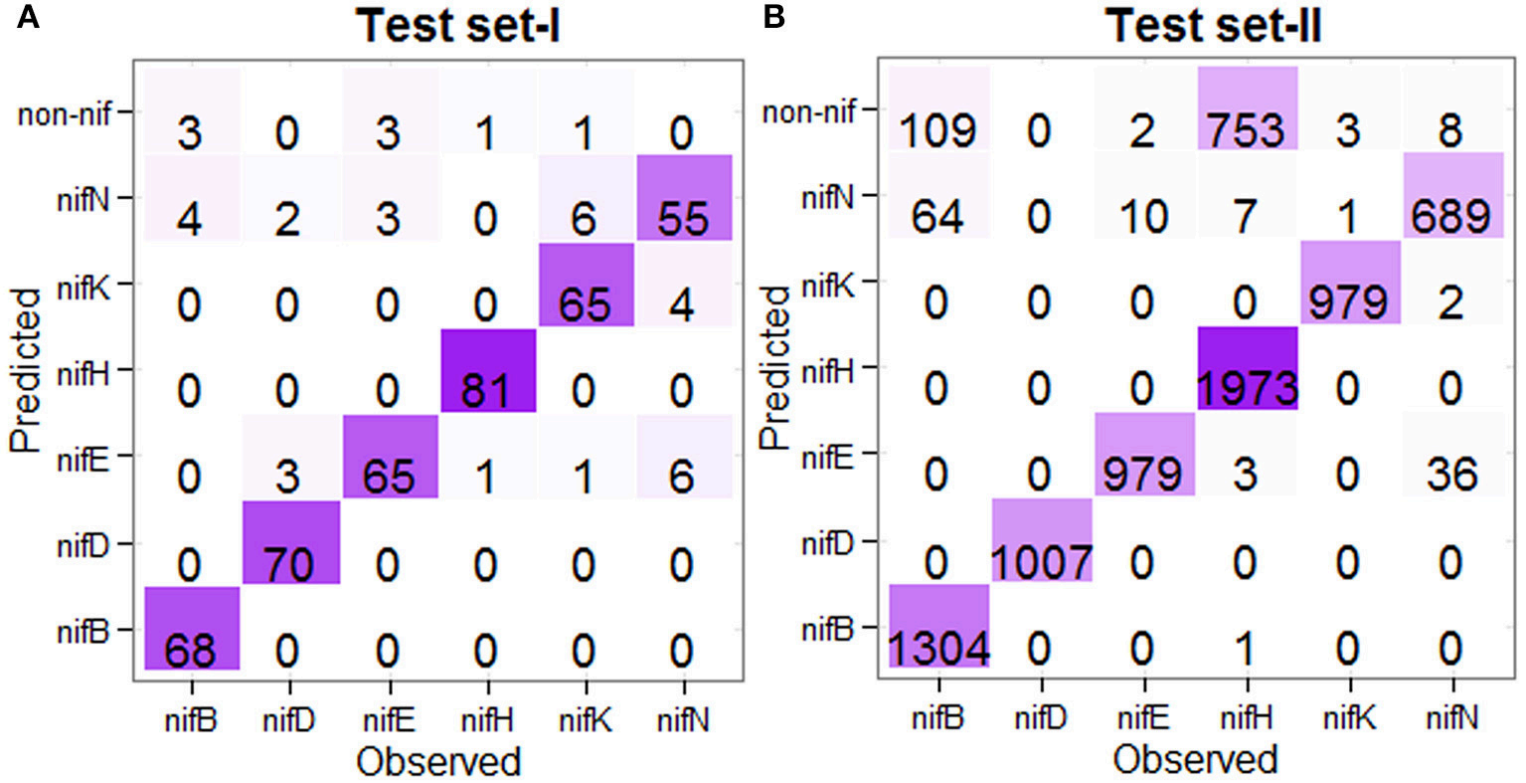
Confusion matrices of the prediction results for the independent datasets. **(A)** Confusion matrix showing the prediction result for the Test set-I, and **(B)** Confusion matrix showing the prediction result for the Test set-II, where the prediction was made in two stages. The color from light to dark represents lower to higher numbers. For the Test set-I, except *nifN* category, >90% sequences are correctly predicted in other five categories. Similarly for the Test set-II, except *nifB* and *nifH*, >90% accuracies are observed in other categories.

For the prediction of test instances, the 438 nif and 438 non-nif protein sequences (as mentioned in the previous subsection) were used to train the model in the first stage, whereas all the nif protein sequences at <90% pair-wise sequence identities (Table 1) were used for training of the model in the second stage. Further, the test sequences which were overlapped with the training sets were also excluded. The number of correctly and wrongly predicted test instances is shown in Figure 5. For the first independent dataset (Test set-I), it is observed that 96% (72/75) of *nifB*, 100% of *nifD*, 95.77% of *nifE*, 98.79% of *nifH*, 98.63% of *nifK*, and 100% of *nifN* are correctly predicted into nif class in the first stage (Figure 5A), whereas 94.44% (68/72), 93.33%, 95.58%, 98.78%, 90.27%, and 84.61% of *nifB, nifD, nifE, nifH, nifK*, and *nifN* respectively are correctly predicted into their corresponding categories in the second stage. Similarly for the second dataset (Test set-II), 92.62% of *nifB*, 100% of *nifD*, 99.78% of *nifE*, 72.48% of *nifH*, 99.69% of *nifK*, and 98.91% of *nifN* are observed to be correctly predicted in the first stage, whereas 95.32% of *nifB*, 100% of *nifD*, 98.98% of *nifE*, 99.44% of *nifH*, 99.89% of *nifK*, and 94.77% of *nifN* are correctly predicted in the second stage (Figure 5B). Similar to the jackknife prediction results (section One-to-one Prediction Analysis), nif protein sequences are mostly misclassified into *nifN* category for both the test datasets, and none of the *nifK, nifE, nifN*, and *nifB* is misclassified into *nifH* and *nifD* categories.

### Proteome-wide prediction analysis

**Figure 6:**
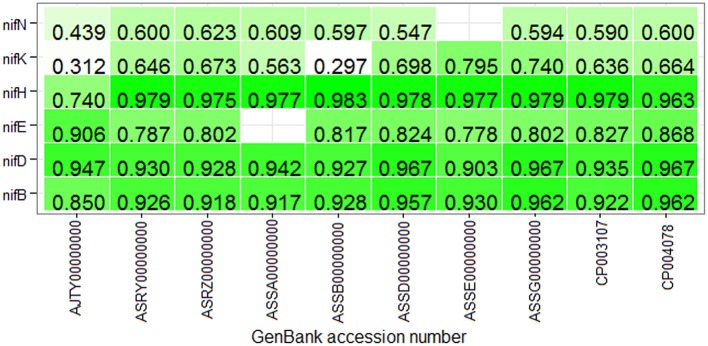
Heat map of the prediction probabilities of nif protein sequences. It shows the probabilities with which the protein sequences of six categories of nitrogen-fixation are predicted in the second stage for the proteome-wide dataset of 10 diaztrophs. The color from light to dark represents lower to higher probabilities, and the blank cell indicates that no sequence was predicted in the corresponding category. Except one *nifE* and one *nifN*, all the nif sequences are correctly predicted. Further with the threshold 0.4, it is observed that except two *nifK* sequences all other nif sequences are correctly predicted in their respective categories.

With the same training dataset (438 nif and 438 non-nif) as mentioned in the previous sub section, ~97% and ~96% of non-nif sequences for non-diaztroph and diaztroph species respectively are observed to be correctly predicted in the first stage (Table 4). Though all the predicted nif sequences (~3%) in non-diaztrophs are false positives, all the sequences predicted as nif in diaztrophs are not false positives. In other words, true positives are also present along with the false positives. Among false positives in both diaztroph and non-diaztroph categories, more number of false positives are seen to be predicted in *nifE, nifN*, and *nifB* as compared to *nifH, nifD*, and *nifK* in the second stage of prediction (Table 4). It is also seen that except one *nifE* of *P. polymyxa* TD94 and one *nifN* of *P. azotofixans* ATCC35681, all other nif sequences are correctly predicted for all the 10 species and with higher probabilities as well (Figure 6). In particular, *nifH, nifD*, and *nifB* are predicted with probabilities >0.9. Also most of the false positives were predicted with <0.4 probabilities (along with only two sequences of *nifK*), and hence the threshold value for prediction of nif protein sequences is set at 0.4 for the second stage with the aim to further reduce the number of false positives. Surprisingly, with this threshold, no *nifH* sequences are predicted in non-diaztroph categoryt except for one species (Table 4). In addition, sequences are also not wrongly predicted into *nifD* and *nifK* category for some non-diaztroph species. Furthermore, the number of false positives which are seen to be higher for *nifE, nifN*, and *nifB* at default threshold is observed to be reduced by ~60% in both diaztroph and non-diaztroph categories (Table 4). It is also found that among the predicted positives in diaztrophs, true positives are obtained with higher probabilities as compared to the false positives, with some exceptions in *nifN* category.

**Table 4:** Performance of the proposed approach for prediction of nitrogen-fixation (nif) proteins using proteome-wide datasets.

**Category**	**Species**	**non-nif**	**nif**											
			**Threshold: default at second stage**						**Threshold: 0.4 at second stage**					
			***nifB***	***nifD***	***nifE***	***nifH***	***nifK***	***nifN***	***nifB***	***nifD***	***nifE***	***nifH***	***nifK***	***nifN***
Non-Diaztroph	*Paenibacillus* sp. JDR 2	6032	19	0	25	3	3	131	8	0	13	0	0	38
Non-Diaztroph	*P*. sp. Y412MC10	6012	24	1	37	1	8	155	8	0	16	0	1	55
Non-Diaztroph	*P.mucilaginosus* KNP414	7582	19	1	40	1	7	154	4	1	24	0	2	59
Non-Diaztroph	*P. mucilaginosus* K02	7137	19	2	41	1	8	146	4	1	22	0	2	59
Non-Diaztroph	*P. mucilaginosus* 3016	6849	21	1	39	2	6	139	5	1	22	0	0	57
Non-Diaztroph	*P. polymyxa* E681	4599	17	3	15	1	4	125	3	1	7	0	2	52
Non-Diaztroph	*P. polymyxa* SC2	4692	14	3	17	0	3	133	2	1	8	0	0	50
Non-Diaztroph	*P. curdlanolyticus* YK9	4662	12	1	24	1	1	114	3	1	13	1	0	53
Non-Diaztroph	*Paenibacillus* sp. HGF5	6275	19	0	35	1	4	162	4	0	14	0	0	56
Non-Diaztroph	*Paenibacillus* sp. HGF7	5816	13	1	27	0	3	132	4	1	14	0	1	40
Diaztroph	*P. polymyxa* TD94	4773	21	4	20	1	3	142	7	2	7	1	3	53
Diaztroph	*P. polymyxa* 1–43	4894	15	3	26	1	5	130	4	2	11	1	3	52
Diaztroph	*P.beijingensis* 1–18	4291	21	2	26	1	6	124	7	1	13	1	3	37
Diaztroph	*Paenibacillus* sp. 1–49	4604	18	3	23	2	4	116	4	2	12	1	1	40
Diaztroph	*P. terrae* HPL-003	5319	20	3	24	1	8	150	4	2	12	1	4	44
Diaztroph	*P. azotofixans* ATCC35681	5005	23	1	25	2	4	150	7	1	13	2	2	59
Diaztroph	*P. graminis* RSA19	5542	17	2	38	1	4	128	5	1	18	1	2	46
Diaztroph	*P. sonchi* X19-5	5792	19	2	37	2	2	131	7	2	20	1	1	44
Diaztroph	*P. zanthoxyli* JH29	4261	18	2	38	2	3	135	5	2	21	2	2	44
Diaztroph	*P. sabinae* T27	4559	23	2	47	5	3	147	9	1	27	5	2	46

*The prediction of nif protein for 10 diaztrophs and 10 non-diaztrophs is made in two stages, where in the first stage the sequences are predicted as nif or non-nif types and the sequences predicted as nif types are only subjected to the second stage in which they are classified into any one of the six categories of nif proteins. In the first stage, classification accuracies are observed >96%. Though, the number of false positives predicted in the second stage are little larger at default threshold, it is reduced by ~60% while predicted with threshold 0.4. Interestingly, no sequences are predicted in nifH category except one species for non-diaztrophs at the threshold 0.4*.

### Prediction analysis with threshold 0.4

As the threshold value 0.4 is found appropriate for prediction of nif sequences in the second stage for 10 different strains of genus *Paenibacillus*, prediction was also made for other species to further validate the determined threshold. For this purpose, protein sequences were collected from four different species viz., *Azoarcus* sp. BH72, *Geofilum rubicundum* JCM15548, *Bacteroides graminisolvens* JCM15093, and *P. Propionicigenes* WB4. We considered these species, because they have already been used in earlier study. The first one is reported in Sarkar and Reinhold-Hurek (2014) and the other two species are reported in Inoue et al. (2015). Moreover, since the true positives were predicted with higher probabilities for most of the times, we considered only the top three predicted positives in each category (*nifH, nifD, nifK, nifE, nifN*, and *nifB*). For *Azoarcus* sp. BH72, all the true nif proteins are predicted with higher probabilities than that of the false ones. In case of *Geofilum rubicundum* JCM15548, *nifH, nifD, nifK*, and *nifE* are predicted with higher probabilities but *nifB* is observed at third place and *nifN* is not predicted correctly. For *Bacteroides graminisolvens* JCM15093, all the nif proteins except *nifN* are predicted correctly as well as with higher probabilities. For *P. Propionicigenes* WB4, all the nif proteins except *nifN* are also predicted correctly and that is with higher probabilities as well. So, it can be said that the threshold value 0.4 can also be useful for prediction of nif proteins in other species as well.

### Online prediction server: nifPred

To help enable experimental scientists, particularly microbiologists working in the area of nitrogen-fixation, an online prediction server nifPred is developed for computational identification of nitrogen fixation proteins. Here, the prediction is made in two stages: (i) the sequences are first classified as nif or non-nif, and (ii) the sequences predicted as nif are only subjected to the second phase, where they are classified into any one of the six nif categories. The nifPred has been trained with 438 nif and 438 non-nif sequences as mentioned in the subsection “Analysis of independent test set prediction” in the first stage, and all the six categories of nif sequences with <90% pair-wise sequence identities (Table 1) in the second stage. The prediction server can be accessed freely at http://webapp.cabgrid.res.in/nifPred. The results are displayed in a tabular format with four columns. The columns from first to fourth respectively represent the serial number, sequence identifier, types of predicted nif proteins, and probabilities with which they are predicted in the corresponding nif categories. Since true nif sequences are predicted with higher probabilities as evidenced from earlier analysis, a maximum of 3 sequences are displayed for any predicted nif category. However, user can download the result for all the supplied sequences from the link “download complete result file” given in the result page. For reproducibility of the proposed work, datasets used to develop the prediction server along with the other datasets used in this work can be obtained from http://webapp.cabgrid.res.in/nifPred/dataset.html. The source code of the nifPred is also made freely available at https://github.com/PrabinaMeher/nifPred.

### Analysis of nifPred

To further assess the efficiency of nifPred, proteome-wide identification of nif proteins was carried out using 49 diaztroph species. The protein sequences for these species were obtained from Uniprot (http://www.uniprot.org/) database. None of the nif proteins of these species were used to train nifPred. It is seen that 34 *nifB*, 49 *nifH*, 49 *nifD*, 49 *nifE*, 42 *nifK*, and 19 *nifN* are correctly identified (Table 5). Further, except one *nifE* and two *nifN* that are correctly predicted with second highest probabilities, all others (34 *nifB*, 49 *nifH*, 49 *nifD*, 48 *nifE*, 42 *nifK*, and 17 *nifN*) are correctly predicted with highest probabilities (Table 5). Though the accuracy is not high for predicting *nifN* sequences, these sequences can be mapped easily on the genome as the six categories of genes occur adjacent to each other. Most importantly, since all the *nifH, nifD*, and *nifE* are correctly identified; identifying other nif gene sequences will not be difficult as they occur in a cluster (Dos Santos et al., 2012). Thus, the nifPred is believed to be an efficient tool for proteome-wide identification of proteins encoded by six categories of nif genes.

**Table 5:** Performance of nifPred in identifying nif proteins in 49 diaztroph species.

**Species**	***nifBB***	***nifDD***	***nifEE***	***nifHH***	***nifKK***	***nifNN***
*Alkaliphilus metalliredigens* QYMF	^#^	^#^	^#^	^#^	^#^	^×^
*Anaeromyxobacter sp*. Fw109-5	^#^	^#^	^*^	^#^	^#^	^×^
*Anaeromyxobacter sp*. K	^#^	^#^	^#^	^#^	^#^	^#^
*Candidatus Accumulibacter phosphatis* clade IIA str. UW-1	^#^	^#^	^#^	^#^	^#^	^#^
*Candidatus Azobacteroides pseudotrichonymphae genomovar*. CFP2	^#^	^#^	^#^	^#^	^#^	^×^
*Chlorobium chlorochromatii* CaD3	^#^	^#^	^#^	^#^	^×^	^#^
*Chlorobium phaeovibrioides* DSM 265	^#^	^#^	^#^	^#^	^×^	^×^
*Chloroherpeton thalassium* ATCC 35110	^#^	^#^	^#^	^#^	^#^	^×^
*Clostridium cellulovorans* 743B	^#^	^#^	^#^	^#^	^#^	^×^
*Denitrovibrio acetiphilus* N2460, DSM 12809	^#^	^#^	^#^	^#^	^#^	^×^
*Desulfatibacillum alkenivorans* AK-01	^×^	^#^	^#^	^#^	^#^	^×^
*Desulfobacca acetoxidans* DSM 11109	^#^	^#^	^#^	^#^	^#^	^×^
*Desulfomicrobium baculatum* DSM 4028	^#^	^#^	^#^	^#^	^#^	^×^
*Desulfotomaculum acetoxidans* DSM 771	^×^	^#^	^#^	^#^	^#^	^×^
*Desulfotomaculum carboxydivorans* CO-1-SRB	^×^	^#^	^#^	^#^	^#^	^×^
*Desulfotomaculum kuznetsovii* DSM 6115	^×^	^#^	^#^	^#^	^#^	^×^
*Desulfotomaculum reducens* MI-1	^×^	^#^	^#^	^#^	^#^	^×^
*Desulfovibrio aespoeensis* Aspo-2	^#^	^#^	^#^	^#^	^#^	^×^
*Desulfovibrio magneticus* RS-1	^#^	^#^	^#^	^#^	^#^	^×^
*Desulfovibrio salexigens* DSM 2638	^#^	^#^	^#^	^#^	^#^	^×^
*Desulfurispirillum indicum* S5	^×^	^#^	^#^	^#^	^×^	^#^
*Desulfurivibrio alkaliphilus* AHT2	^×^	^#^	^#^	^#^	^×^	^#^
*Dickeya dadantii* Ech703	^×^	^#^	^#^	^#^	^#^	^#^
*Erwinia carotovora atroseptica* SCRI1043	^×^	^#^	^#^	^#^	^#^	^#^
*Ethanoligenens harbinense* YUAN-3	^#^	^#^	^#^	^#^	^#^	^#^
*Geobacter bemidjiensis* Bem	^#^	^#^	^#^	^#^	^#^	^#^
*Hydrogenobacter thermophilus* TK-6	^×^	^#^	^#^	^#^	x	^#^
*Hyphomicrobium sp*. MC1	^#^	^#^	^#^	^#^	^#^	^#^
*Ilyobacter polytropus* CuHBu1, DSM 2926	^#^	^#^	^#^	^#^	^#^	^×^
*Leptothrix cholodnii* SP-6	^#^	^#^	^#^	^#^	^#^	^×^
*Methanoplanus petrolearius* SEBR 4847, DSM 11571	^×^	^#^	^#^	^#^	^#^	^×^
*Methanosaeta concilii* GP6	^×^	^#^	^#^	^#^	^#^	^×^
*Methylacidiphilum infernorum* V4	^#^	^#^	^#^	^#^	^#^	^#^
*Paludibacter propionicigenes* WB4	^#^	^#^	^#^	^#^	^#^	^×^
*Pectobacterium atrosepticum* SCRI1043	^×^	^#^	^#^	^#^	^#^	^#^
*Pelobacter carbinolicus* DSM 2380	^#^	^#^	^#^	^#^	^#^	^×^
*Pelodictyon luteolum* DSM 273	^#^	^#^	^#^	^#^	^#^	^×^
*Pelodictyon phaeoclathratiforme* BU-1	^#^	^#^	^#^	^#^	^#^	^×^
*Sideroxydans lithotrophicus* ES-1	^#^	^#^	^#^	^#^	^#^	^#^
*Spirochaeta smaragdinae* SEBR 4228, DSM 11293	^×^	^#^	^#^	^#^	^#^	^×^
*Sulfuricurvum kujiense* DSM 16994	^#^	^#^	^#^	^#^	^#^	^*^
*Syntrophobacter fumaroxidans* MPOB	^#^	^#^	^#^	^#^	^#^	^×^
*Syntrophobotulus glycolicus* DSM 8271	^#^	^#^	^#^	^#^	^×^	^×^
*Thermincola potens* JR	^#^	^#^	^#^	^#^	^#^	^×^
*Thermocrinis albus* HI 11/12, DSM 14484	^#^	^#^	^#^	^#^	^×^	^#^
*Thermodesulfovibrio yellowstonii* DSM 11347	^#^	^#^	^#^	^#^	^×^	^#^
T*hermosaccharolyticum* DSM 571	^×^	^#^	^#^	^#^	^#^	^×^
*Tolumonas auensis* DSM 9187	^#^	^#^	^#^	^#^	^#^	^*^
*Zymomonas mobilis mobilis* ZM4	^#^	^#^	^#^	^#^	^#^	^#^

*To assess the performance of nifPred with threshold value 0.4, prediction for nif proteins is made by using proteome-wide datasets of diaztrophs. The nifH, nifD, and nifE are correctly predicted in all the 49 species. Besides, 42 nifK, 34 nifB, and 19 nifN are also correctly identified in 49 species*.

× , wrongly predicted;

# , predicted with highest probability;

* *, predicted with second highest probability*.

## Discussion

The study regarding functional genomics and proteomics of diaztrophs has great importance in twenty-first century (Sur et al., 2010). The works related to the genetics of nif was first initiated in *Klebseilla*, where organization of nif genes were reported (Arnold et al., 1988). Thereafter, many studies (Dixon and Kahn, 2004; Hu et al., 2007; Rubio and Ludden, 2008) have established that several nif genes are necessary for BNF by diaztrophs. In particular, Dos Santos et al. (2012) proposed the criteria of co-occurrence of six genes set (*nifB, nifD, nifE, nifH, nifK*, and *nifN*) for the characterization of nitrogen fixing microbes. Although BNF is confined to a subset of prokaryotes, species-specific environmental, and metabolic conditions are essential for the manifestation of biochemical pathways, and thus obtaining a comprehensive census of this trait is not easy (O'Carroll and Dos Santos, 2011). However, voluminous structural information generated with the development of efficient bioinformatics tools as well as proteomics technologies guaranteed the acceleration in nitrogen-fixation (nif) research (Sur et al., 2010). In this regard, the current study presents a computational tool for the identification of protein sequences encoded by the six categories of nif genes.

Six different kinds of sequence-based features viz., AAC, DPC, PseAAC, CTD, GPC, and ACF were considered for mapping the protein sequences onto numeric feature vectors, which were then supplied to SVM as input for classification using the four widely used kernel functions. Actually, two types of classifications were made viz., binary and multi-class. In binary classifier, classification was made between nif and non-nif proteins, where the classification accuracy was found to be higher for the combination of CTD features and radial kernel as compared to the other feature-kernel combinations. Using this feature-kernel combination, binary classification was also carried out with six other machine learning classifiers viz., RF, ANN, NB, kNN, Bagging and Boosting. From the results of comparison, classification accuracies were not only found to be higher for SVM but also most stable as compared to the other classifiers. Using the same feature-kernel combination, multi-class classification was further carried out for classification of six categories of nif proteins encoded by *nifB, nifD, nifE, nifH, nifK*, and *nifN* genes. Classification accuracies were found to be higher for *nifH* and *nifD* categories, whereas lowest for *nifN* category. Specifically, the sequences were mostly misclassified into *nifN*, whereas no sequence was misclassified into *nifH* and only one sequence was misclassified into *nifD* category. This implies that the sequences of *nifH* and *nifD* share a higher degree of similarity within the classes and lesser degree of similarity with other nif categories, whereas the sequences of *nifN* share a lesser degree of similarity within the classes. This similarity results can also be inferred from the CD-HIT analysis. The higher degree of similarity within *nifH* may be the reason that the *nifH* sequences have been successfully used as markers for biodiversity study of diaztrophs through phylogenetic analysis (Dos Santos et al., 2012).

No computational approach is available for the prediction of nif genes related to BNF, and on the other hand the blast algorithms are useful for finding protein homologs. Hence, the performance of the developed computational model was compared with that of homology based method i.e., BlastP and PSI-Blast algorithms. Both the algorithms were found to be highly biased toward the positive class. In other words, number of false positives were found much higher than that of proposed approach. Moreover, blast algorithm depends upon sequence alignment and may take more time for large number of sequences. Besides, the performance of the proposed model was also compared with that of HMM with e-values 1 and 10, where the proposed approach was found to achieve higher accuracies in terms of all the considered performance metrics. Further, when HMM was compared with blast algorithms, the overall accuracy of the HMM was found higher than that of blast algorithms. The proposed computational model was also evaluated with independent datasets. Despite being trained with small number of sequences at both stages, accuracies were found to be >90% in both stages. This implies that the model was well-trained with the CTD features and radial kernel of SVM.

Since each *nifB, nifD, nifE, nifH, nifK*, and *nifN* genes are likely to be present in each diaztroph once (with certain exception in case of gene duplication); prediction of these sequences from whole genome data is very much challenging. Thus, the developed methodology was also evaluated with proteome-wide datasets of 10 diaztroph and 10 non-diaztroph species. With the threshold value 0.4 at the second stage, *nifH* of all diaztrophs were correctly predicted with 1 or 2 false positives. On the other hand, no *nifH* sequences were predicted in non-diaztrophs barring one species. Besides, a maximum of 1 or 2 false *nifD* and *nifK* sequences were found in both diaztrophs and non-diaztrophs. However, in case of diaztrophs, the true positives were predicted with higher probabilities as compared to the false positives. It was also found that the number of false positives were higher for the biosynthetic components (*nifE, nifN*, and *nifB*) as compared to the catalytic components (*nifH, nifD*, and *nifK*). Therefore, it can be said that with a very few number of false positives nif sequences can be correctly predicted using proteome-wide datasets. Since no *nifH* sequences were predicted in nine out of the 10 non-diaztrophs, it can be said the there is a possibility of getting homologs of other nif sequences except *nifH*. Thus, the proposed approach can be used to predict the potential diaztrophs and supplement the transcriptome profiling and comparative genomic approaches for identifying nif genes in nitrogen fixing microbes.

In addition to the Mo-Fe nitrogenese (Nif), two types of alternative nitrogenase have also been reported in literature i.e., Vanadium dependent nitrogenase (Vnf) and Iron-only nitrogenase (Anf). These Nif, Vnf, and Anf types of nitrogenase are homologus and evolutionary related (Raymond et al., 2004; Inoue et al., 2015). Despite differences in their metal content, these nitrogenase types have common structural and mechanistic features. The Fe-Mo nitrogenase has been reported to be intrinsically more efficient in nitrogen reduction than either of the nitrogenase (Joerger and Bishop, 1988; Miller and Eady, 1988). Since the conservation in primary sequences for all the three types of nitrogenase are similar, the proposed approach may also be useful for predicting the proteins encoded by Vnf and Anf genes.

Based on the developed methodology, an online prediction server nifPred has also been established for the identification of six categories of nif proteins. The server not only represents the future direction for developing other computational methods (Shen, 2009), but is also important for most of the experimental scientists working in the field of nitrogen fixation. The nifPred can be easily used by the researchers for proteome-wide identification of nif proteins encoded by *nifB, nifD, nifE, nifH, nifK*, and *nifN* genes, without going into details of the statistical methods adopted in developing the approach. Since accuracy is less as far as *nifN* is concerned, effort will be put in future for further improvement in identifying *nifN* genes encoded proteins.

In the current study, we propose the first methodology to computationally identify the six categories of nitrogen fixation proteins, which are assumed to be essential for any diaztroph to fix the atmospheric nitrogen into ammonia. The developed web server is expected to supplement the transcriptional profiling and comparative genomics studies for the identification and functional annotation of genes related to BNF. The server will not only be useful for identification of diaztroph and non-diaztroph species, but also support for the functional annotation of nif genes on the genome of many nitrogen fixing microbes.

## Author contributions

PM, AR, and MG conceived and designed the study. JM, TS, SG, PM, and SP collected and analyzed the sequence dataset. PM developed the prediction approach. TS and PM developed the web server. PM, JM, SP, and SG drafted the manuscript. PM, TS, AR, JM, SG, SP, and MG corrected and refined the manuscript. All authors read and approved the final manuscript.

### Conflict of interest statement

The authors declare that the research was conducted in the absence of any commercial or financial relationships that could be construed as a potential conflict of interest.
