# Supplementary material for: nifPred: Proteome-Wide Identification and Categorization of Nitrogen-Fixation Proteins of Diaztrophs Based on Composition-Transition-Distribution Features Using Support Vector Machine
Source: Front Microbiol. 2018 May 29;9:1100. doi: 10.3389/fmicb.2018.01100 (PMC5986947; doi:10.3389/fmicb.2018.01100)
Supplement: Supplementary file 1 [file Table_1.DOC]

### Supplementary Table S1

### Summary of the independent dataset. The two independent datasets viz., Test set-I and Test set-II are prepared by collecting the protein sequences from the study of Do Santos et al. (2012) and InterPro ([**https://www.ebi.ac.uk/interpro/**](https://www.ebi.ac.uk/interpro/)) database respectively.

| **Dataset** | ***nifH*** | ***nifD*** | ***nifK*** | ***nifE*** | ***nifN*** | ***nifB*** |
| --- | --- | --- | --- | --- | --- | --- |
| Test set-I | 83 | 75 | 73 | 71 | 65 | 75 |
| Test set-II | 2737  (IPR005977) | 1007  (IPR005972) | 983  (IPR005976) | 991  (IPR005973) | 735  (IPR005975) | 1477  (IPR005980) |

### The InterPro id for the Test set-II is provided inside the bracket.
