# Supplementary material for: nifPred: Proteome-Wide Identification and Categorization of Nitrogen-Fixation Proteins of Diaztrophs Based on Composition-Transition-Distribution Features Using Support Vector Machine
Source: Front Microbiol. 2018 May 29;9:1100. doi: 10.3389/fmicb.2018.01100 (PMC5986947; doi:10.3389/fmicb.2018.01100)
Supplement: Supplementary file 2 [file Table_2.DOC]

**Supplementary Table S2**

Summary of the proteome-wide datasets. The protein sequences for 10 diaztroph and 10 non-diaztroph species are collected from NCBI (<https://www.ncbi.nlm.nih.gov/>). These datasets are used to evaluate the performance of proposed approach in proteome-wide identification of nitrogen-fixation proteins encoded by six categories of genes viz., *nifH*, *nifD*, *nifK*, *nifE*, *nifN* and *nifB*.

| **Category** | **Species** | **Gene Bank Accession** | **#Sequence*** |
| --- | --- | --- | --- |
| Non-Diaztroph | *Paenibacillus* sp. JDR 2 | CP001656 | 6213 |
| Non-Diaztroph | *Paenibacillus* sp. Y412MC10 | CP001793 | 6238 |
| Non-Diaztroph | *P.mucilaginosus* KNP414 | CP002869 | 7804 |
| Non-Diaztroph | *P. mucilaginosus* K02 | CP003422 | 7354 |
| Non-Diaztroph | *P. mucilaginosus* 3016 | CP003235 | 7057 |
| Non-Diaztroph | *P. polymyxa* E681 | CP000154 | 4764 |
| Non-Diaztroph | *P. polymyxa* SC2 | CP002213 | 4862 |
| Non-Diaztroph | *P. curdlanolyticus* YK9 | AEDD00000000 | 4815 |
| Non-Diaztroph | *Paenibacillus* sp. HGF5 | AEXS00000000 | 6496 |
| Non-Diaztroph | *Paenibacillus* sp. HGF7 | AFDH00000000 | 5992 |
| Diaztroph | *P. polymyxa* TD94 | ASSA00000000 | 5697 |
| Diaztroph | *P. polymyxa* 1–43 | ASRZ00000000 | 5731 |
| Diaztroph | *P.beijingensis* 1–18 | ASSB00000000 | 5599 |
| Diaztroph | *Paenibacillus* sp. 1–49 | ASRY00000000 | 5628 |
| Diaztroph | *P. terrae* HPL-003 | CP003107 | 5525 |
| Diaztroph | *P. azotofixans* ATCC35681 | ASSE00000000 | 5722 |
| Diaztroph | *P. graminis* RSA19 | ASSG00000000 | 7081 |
| Diaztroph | *P. sonchi* X19-5 | AJTY00000000 | 7705 |
| Diaztroph | *P. zanthoxyli* JH29 | ASSD00000000 | 5622 |
| Diaztroph | *P. sabinae* T27 | CP004078 | 5250 |

*Number of sequences are obtained after removing the sequences with non-standard residues as well as the sequences those are overlapped with training set as mentioned in sub-section “analysis of proteome-wide prediction” of “Results” section
